# Tripartite Motif-Containing Protein 30 Modulates TCR-Activated Proliferation and Effector Functions in CD4^+^ T Cells

**DOI:** 10.1371/journal.pone.0095805

**Published:** 2014-04-22

**Authors:** Un Yung Choi, Ji Yeon Hur, Myeong Sup Lee, Quanri Zhang, Won Young Choi, Lark Kyun Kim, Wook-Bin Lee, Goo Taeg Oh, Young-Joon Kim

**Affiliations:** 1 Department of Biochemistry, College of Life Science and Technology, Yonsei University, Seoul, Korea; 2 Department of Integrated Omics for Biomedical Science, WCU Program of Graduate School, Yonsei University, Seoul, Korea; 3 Division of Life and Pharmaceutical Sciences, Ewha Women’s University, Seoul, Korea; Yonsei University, Republic of Korea

## Abstract

To avoid excessive activation, immune signals are tightly controlled by diverse inhibitory proteins. TRIM30, a tripartite motif (TRIM)-containing protein is one of such inhibitors known to function in macrophages. To define the roles of TRIM30, we generated *Trim30* knockout (*Trim30* ^−/−^) mice. *Trim30* deletion caused no major developmental defects in any organs, nor showed any discernable defect in the activation of macrophages. But, *Trim30* ^−/−^ mice showed increased CD4/CD8 ratio when aged and *Trim30* ^−/−^ CD4^+^ T cells exhibited an abnormal response upon TCR activation, in particular in the absence of a costimulatory signal. Adoptive transfer of wild-type and *Trim30* ^−/−^ CD4^+^ T cells together into lymphopenic hosts confirmed higher proliferation of the *Trim30* ^−/−^ CD4^+^ T cells *in vivo*. Despite the enhanced proliferation, *Trim30* ^−/−^ T cells showed decreased levels of NF-κB activation and IL-2 production compared to wild-type cells. These results indicate a distinct requirement for TRIM30 in modulation of NF-κB activation and cell proliferation induced by TCR stimulation.

## Introduction

Lymphocyte proliferation is important for proper response to infection, but uncontrolled proliferation predisposes individuals to lymphoproliferative disorders (LDs) or autoimmune disease [1], [2]. Therefore, peripheral T cells exhibit inhibitory mechanisms to control the two major signals of T cell activation: the MHC-TCR interaction and the CD28 costimulation. Several E3 ligases including Protein pellino homolog 1 (PELI1), Gene Related to Anergy In Lymphocytes (GRAIL), Casitas B-lineage lymphoma b (Cbl-b) appear to play important roles in the downregulation of TCR signals [3]. PELI1 negatively regulates c-Rel by mediating ubiquitination of Lys48 during T-cell activation [4]. Casitas B-lineage lymphoma b (Cbl-b) negatively regulates GDP/GTP exchange factor Vav1 phosphorylation [5], while GRAIL promotes CD3 receptor ubiquitination, leading to degradation [6]. Knockout of these E3 ligases in mice results in a high susceptibility to autoimmune diseases [4], [7], [8] in these animals, indicating that E3 ligase-mediated degradation of TCR signaling molecules is a major inhibitor of T cell activation.

The tripartite motif-containing TRIM [also called ring-B box-coiled coil motif (RBCC)] proteins belong to the RING-type E3 ligase family [9], and many of these ligases have been implicated in regulation of the immune response [10], [11]. Human TRIM5α acts as a retroviral restriction factor that recognizes retroviral capsids [12] and promotes innate immune signaling [13], [14]. Mouse TRIM30, a human TRIM5α homolog, was shown to downregulate TLR-mediated NF-κB signaling by targeting TAK1-binding protein 2 (TAB2) and TAK1-binding protein 3 (TAB3) for degradation using siRNA-mediated knockdown experiments [15]. Downregulation of NACHT, LRR, and PYD domains-containing protein 3 (NLRP3) during inflammasome activation by reactive oxygen species also requires TRIM30 [16].

In addition to these regulatory function of TRIM30 in macrophages and dendritic cells, high levels of TRIM30 expression are observed in diverse lymphocyte populations [15] and are inversely related to IL-2R expression in T cells [17]; however, whether TRIM30 controls the NF-κB pathway in lymphocytes is not known. To determine the physiological function of TRIM30 and its role in lymphocytes, we generated *Trim30* knockout mice. Interestingly, we found no major defects in the activation of macrophages, but *Trim30* ^−/−^ CD4^+^ T cells exhibited enhanced proliferation and cell cycle progression upon TCR stimulation both *in vitro* and *in vivo* compared to wild-type cells; however, CD4^+^ T cell activation mediated by CD3 stimulation was compromised, particularly with regard to the activation of NF-κB signaling. These findings revealed distinct roles for TRIM30 during the activation of naïve T cells upon TCR stimulation.

## Results

### Abnormal Age-associated CD4/CD8 Ratio in *Trim30^−/−^* Mice

**Figure 1:**
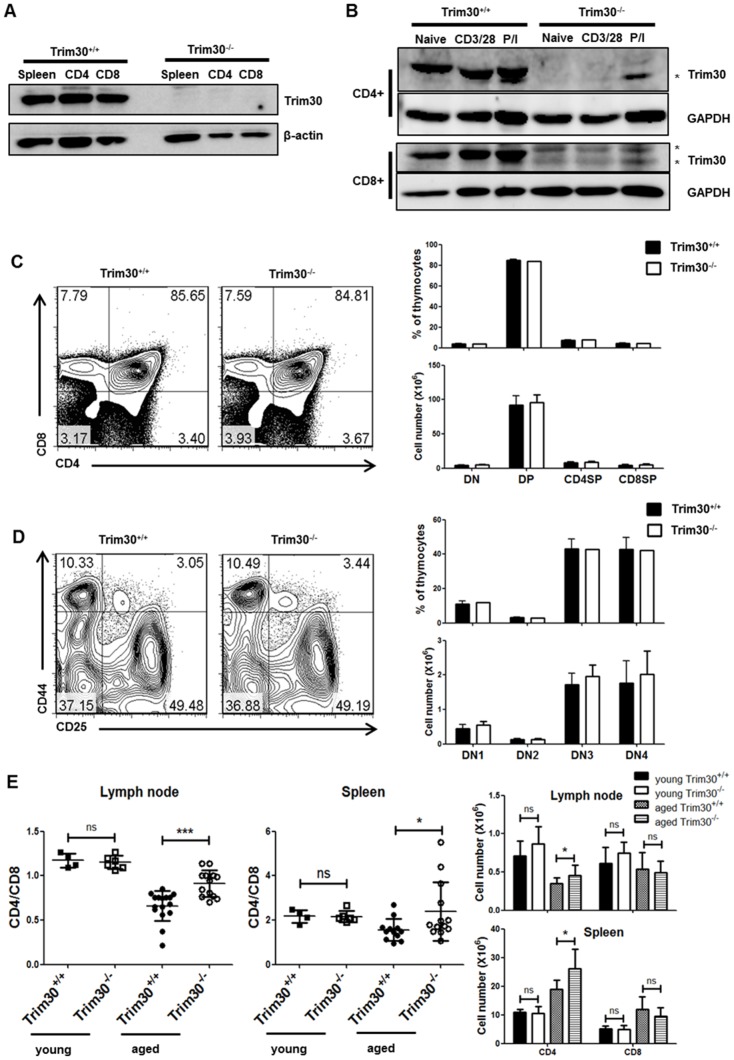
Generation of *Trim30* knockout mice. (**A**) A diagram representing the targeting construct, the *Trim30* gene locus (Wild-type locus), and the locus after targeting (Targeted locus). The targeting construct contains a stop codon and a neomycin selectable marker in exon 2 of *Trim30*. (**B**) Genomic DNA fragments from *Trim30* ^+/−^ and *Trim30* ^−/−^ progeny after Southern blotting with NcoI digestion. Wild-type alleles (10 kb) and the targeted alleles (8 kb) are indicated. (**C**) Genomic DNA isolated from *Trim30* ^+/+^, *Trim30* ^−/−^, and *Trim30* ^+/−^ was subjected to PCR. (**D**) Tissue *Trim30* mRNA expression from *Trim30* ^+/+^ and *Trim30* ^−/−^ mice. RT-PCR analysis revealed high *Trim30* transcript levels in lymphoid organs (spleen, thymus, and lymph node) and bone marrow in contrast to the low levels of *Trim30* transcripts in non-hematopoietic tissues (**E**) TRIM30 protein expression level in tissues from *Trim30* ^+/+^ and *Trim30* ^−/−^ mice as determined with immunoblotting using anti-Trim30 antibody. *, non-specific signal. (**F**) BMDMs were stimulated with LPS (200 ng/ml) or poly(I:C) (5 µg/ml), and *Trim30* transcripts were quantified by quantitative RT-PCR. For detection of cytokine expression, *Trim30^+/+^* and *Trim30^−/−^* BMDMs were pretreated for 18 hr with LPS (LSP pre) and then restimulated with LPS (LPS re) indicated time or stimulated with poly(I:C) and transcripts for indicated cytokines were quantified by quantitative RT-PCR. Expression was normalized to GAPDH. (**G**) Survival of mice (n = 14 per group) given i.p injection of LPS (20 mg/kg) (upper panel). Survival of mice (n = 18 per group) given i.p infection of Listeria monocytogenes (2×10^6^ CFU per mouse) (lower panel). Data are representative results from three independent experiments. Error bars in D, E, F indicate s.d.

To understand the physiological function of TRIM30, we generated *Trim30* knockout mice. The second exon, which contains the start codon, was replaced with a neomycin selection cassette following a stop codon (Figure 1A), and the targeted construct was germ-line transformed to generate chimeric *Trim30* ^+/−^ mice, which were confirmed both by Southern blotting- and PCR-based genotyping (Figure 1B–C). The intercross of the *Trim30* ^+^/− heterozygotes produced offspring at the expected Mendelian ratio, indicating that the mutants experienced no obvious developmental defects associated with *Trim30* deletion. RT-PCR analysis revealed high *Trim30* transcript levels in lymphoid organs (spleen, thymus, and lymph node) and bone marrow in contrast to the low levels of *Trim30* transcripts in non-hematopoietic tissues (Figure 1D). The high levels of basal and induced expression of *Trim30* in lymphocytes and macrophages were absent in the *Trim30* knockout mouse. Immunoblot analysis of various tissues also confirmed the loss of TRIM30 protein expression in the lymph nodes, spleen, and thymus of *Trim30* ^−/−^ mice (Figure 1E).

To validate its suggested role in NF-kB activation in macrophages, Trim30^+/+^ and Trim30^−/−^ bone marrow derived macrophages (BMDMs) were challenged with LPS or poly I:C then compared for their cytokine responses. The challenge with TLR ligands induced TRIM30 strongly only in wild-type cells, but there was no discernable difference in the expression of the major cytokines (*IL-6, TNFα, IL-12 p40, IFNα, IFNβ*) (Figure 1F). Besides, Trim30^−/−^ mice showed no defect in resistance to LPS-induced septic shock, as well as in the response to *Listeria monocytogenes* infection (Figure 1G). Therefore, TRIM30 appears dispensable for most TLR activations in macrophages.

**Figure 2:**
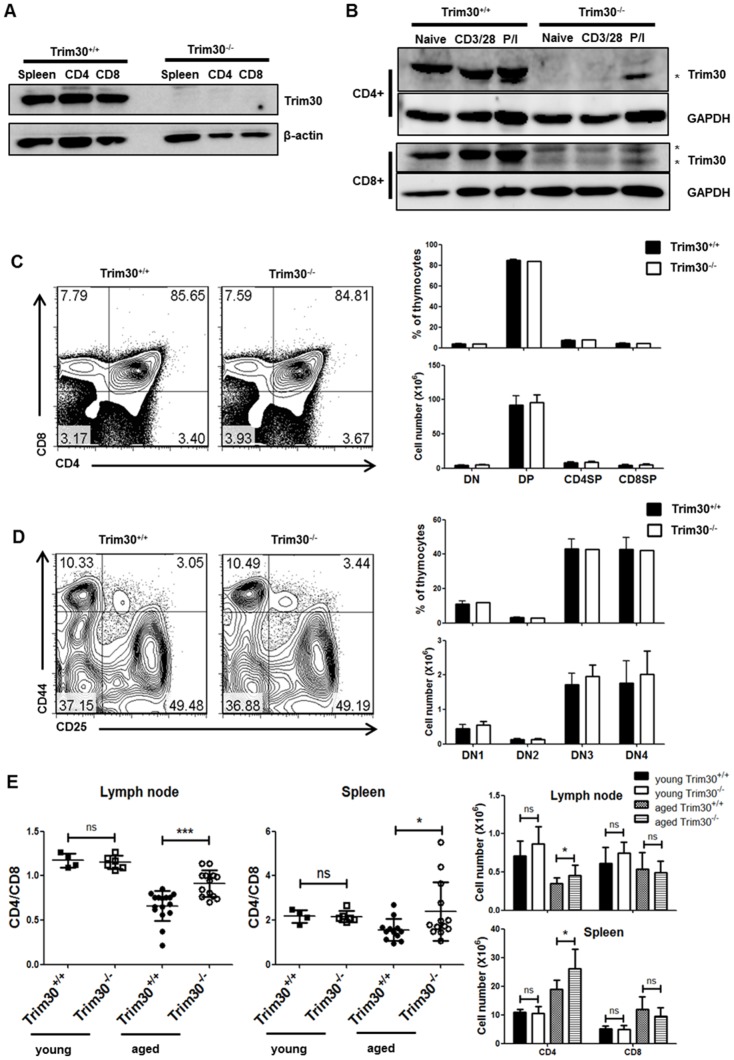
T cell lymphoid organ developmental progression is normal in *Trim30* knockout mice. Immunoblot analysis of Trim30 expression in splenocytes and purified CD4^+^ and CD8^+^ T cells that were (**A**) unstimulated or, β-actin was used as a loading control (B) stimulated with anti-CD3 (2 µg/ml) and anti-CD28 (2 µg/ml) antibodies (CD3/CD28) or with 10 ng/ml of PMA and 500 ng/ml of ionomycin (P/I) for 3 days. GAPDH was used as a loading control. (**C**) Representative flow cytometric plots for CD4 and CD8 expression in the thymocyte population from *Trim30* ^+/+^ and *Trim30* ^−/−^mice. The average percentages and absolute cell number of CD4^−^CD8^−^ double negative (DN), CD4^+^CD8^+^ double positive (DP), and CD4^+^ or CD8^+^ single positive (CD4SP, CD8SP) thymocytes from four independent experiments are shown on the right. (D) Representative flow cytometric plots for CD44 and CD25 expression in the identified DN1 (CD44^+^CD25^−^), DN2 (CD44^+^CD25^+^), DN3 (CD44^−^CD25^+^), and DN4 (CD44^−^CD25^−^) T cell populations. DN1-DN4 thymic populations and absolute cell number from four experiments are shown on the right. (E) Ratios of CD4/CD8 on lymph node cells and splenocytes isolated from 10-week- (young) and 12-month-old (aged) *Trim30* ^+/+^ and *Trim30* knockout mice. For this analysis, at least four young mice or 12 aged mice were analyzed. Absolute cell number of independent experiment are shown on the right. The *P*-value was calculated using a *t*–test. *, *P*<0.05; ***, *P*<0.0005. Error bars in C, D, and E represent the s.d.

In contrast to the inducible expression of *Trim30* in macrophages, the high basal levels observed in lymphoid organs suggest that TRIM30 protein may be involved in the regulation of lymphocytes. To this end, we first assessed TRIM30 expression in T cells. Immunoblot analysis revealed that TRIM30 is highly expressed in both CD4^+^ T cells and CD8^+^ T cells purified from wild-type spleens (Figure 2A). TRIM30 is abundant in the naïve T cells, and high levels of TRIM30 were maintained after T cell activation with anti-CD3/CD28 antibodies or PMA/ionomycin costimulation (Figure 2B). Comparison of T lymphocyte populations in thymus from *Trim30* ^+/+^ and *Trim30* ^−/−^ mice revealed no differences in the proportions of double-negative (DN), double-positive (DP), CD4 single-positive (SP), or CD8SP thymocytes (Figure 2C). In addition, the four DN populations, which were sorted based on the expression of CD25 and CD44, were comparable in *Trim30* ^+/+^ and *Trim30* ^−/−^ mice (Figure 2D). Therefore, the development of T cells appeared normal in the *Trim30* mutant mice. However, comparison of aged mice revealed significant difference in the ratios of peripheral CD4/CD8 T cells (Figure 2E). As mice age, the relative ratio between CD4^+^ and CD8^+^ T cells gradually decreases [18], [19]; however, in aged *Trim30* ^−/−^ mice, the CD4/CD8 ratio remained high in the spleen and lymph nodes. Absolute number of CD4^+^ T cells was slightly increased in *Trim30* ^−/−^. Difference in CD4/CD8 ratio was due to more abundant CD4^+^ T cells in *Trim30* ^−/−^ mice. These finding suggest that long-term peripheral maintenance of T cells is dysregulated in *Trim30* ^−/−^ mice.

### Hyper-proliferation of *Trim30^−/−^* CD4^+^ T cells

**Figure 3:**
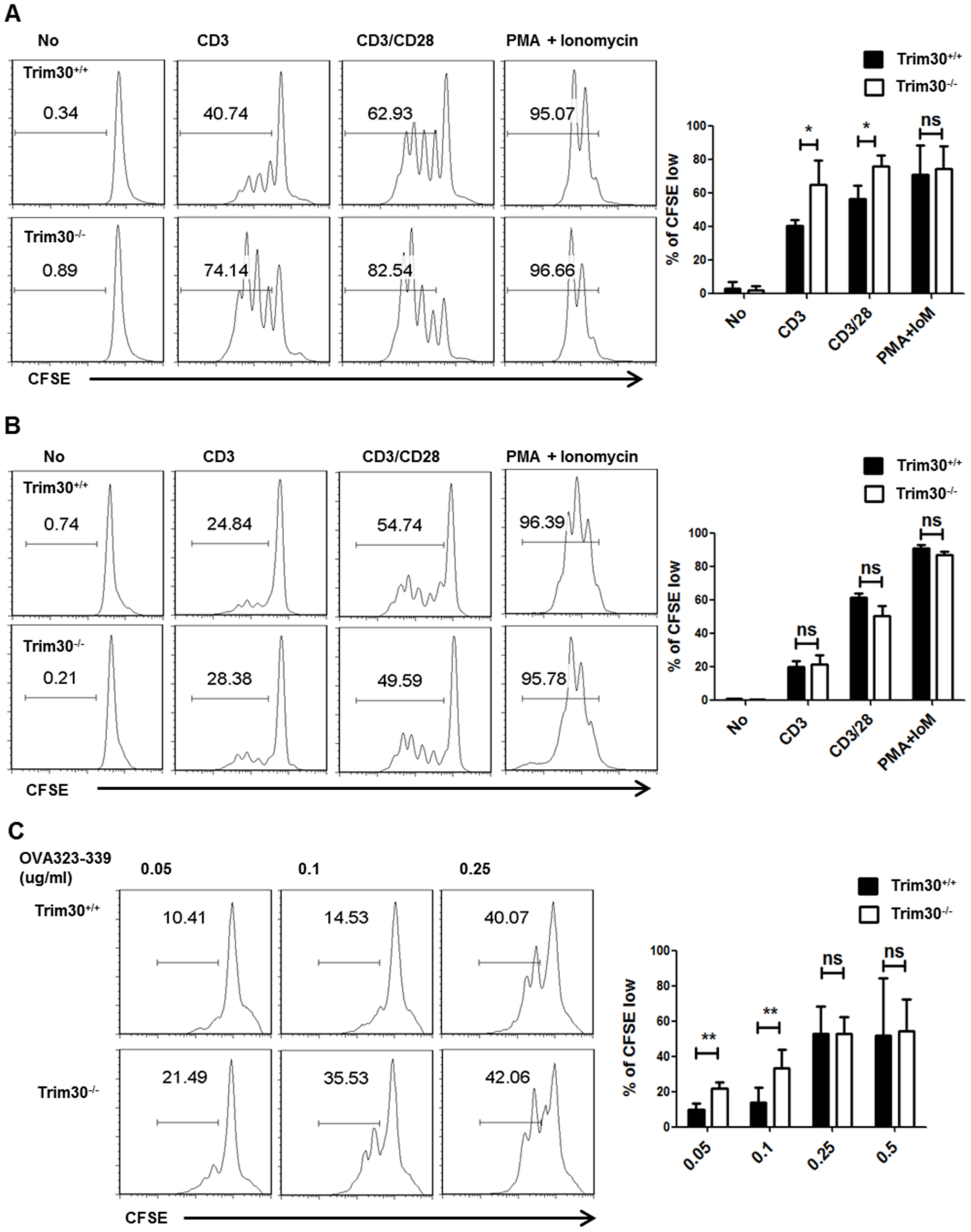
Enhanced proliferation of *Trim30* ^−/−^ CD4 T cells. (**A**) Purified CD4^+^ T cells from *Trim30* ^+/+^ and *Trim30* ^−/−^ spleens were labeled with CFSE and cultured with the indicated supplements (No, medium; CD3, 2 µg/ml of plate-coated anti-CD3 antibody; CD3/CD28, 2 µg/ml of plate-coated anti-CD3 antibody and soluble anti-CD28 antibody; PMA+Ionomycin, 10 ng/ml of PMA and 500 ng/ml of ionomycin) for 3 days. The CFSE intensity of the CD4^+^ T cells was measured with flow cytometry, and representative plots for *Trim30* ^+/+^ and *Trim30*−/− T cells are shown with the percentages of the dividing cells given as those with diluted levels of CFSE staining. (**B**) Purified CD8^+^ T cells from *Trim30* ^+/+^ or *Trim30* ^−/−^ mouse spleens were analyzed as described above for (A). (**C**) Splenocytes from *Trim30* ^+/+^and *Trim30* ^−/−^OT-II mice were activated with the indicated concentration of Ova 323–339 peptide for 3 days. Proliferation of T cells was measure by CFSE intensity after gating for CD4^+^ cells. The results of four independent measurements are shown on the right. The *P*-value was calculated using the *t*–test. *; *P*<0.05; **, *P*<0.005, ns; not significant. Error bars represent s.d.

We further investigated the role of TRIM30 in the response of CD8^+^ and CD4^+^ T cells in vitro. We labeled purified *Trim30* ^+/+^ and *Trim30* ^−/−^ splenic T cells with the division-tracking dye CFSE, and then stimulated the cells for 72 h with anti-CD3 antibody in the presence or absence of costimulation with anti-CD28 antibody. Upon stimulation with anti-CD3 agonist antibody, 74% of the *Trim30* ^−/−^ CD4^+^ T cells proliferated, while only 40% of the CD4^+^ T cells proliferated in wild type mice (Figure 3A). The proliferation disparity between *Trim30* ^+/+^ and *Trim30* ^−/−^ CD4^+^ T cells decreased upon stronger stimulation in the presence of costimulatory signal. When cells were stimulated with PMA and ionomycin, which bypass TCR signaling, proliferation of *Trim30* ^−/−^ cells was comparable to that of *Trim30* ^+/+^cells. These hyper-proliferation defects appeared specific to CD4^+^ T cells because CD8^+^ T cells from *Trim30* ^−/−^ mice proliferated normally in response to these stimuli (Figure 3B). Therefore, TRIM30 seemingly represses premature CD4^+^ T cell proliferation, particularly in the absence of a costimulatory signal for TCR activation.

Subsequently, we determined whether hyper-proliferation of *Trim30* ^−/−^ CD4^+^ T cells is also observed during antigen-specific T cell stimulation. To this end, we isolated splenocytes from *Trim30* ^−/−^ mice crossed with OT-II transgenic mice. Consistent with the above results, *Trim30* ^−/−^ OT-II CD4^+^ T cells exhibited 10–20% enhanced proliferation compared with *Trim30* ^+/+^ OT-II CD4^+^ T cells, particularly when stimulated with a low dose of OVA_323–339_ peptide (Figure 3C). We cannot rule out the possibility of that loss of Trim30 might also compromise antigen presenting cells function. Nevertheless, we detected a normal cytokine expression in Trim30^−/−^ macrophage, a type of antigen presenting cells (Figure 1F), and the hyper-proliferation was more clear when only CD4^+^ T cells were used (Figure 3A). Overall, these data indicate that TRIM30 negatively regulates CD4^+^ T cell proliferation following TCR activation in the absence of costimulatory signaling or low levels of TCR-specific antigens.

### Increased Cell Cycle Progression in *Trim30* Knockout T cells

**Figure 4:**
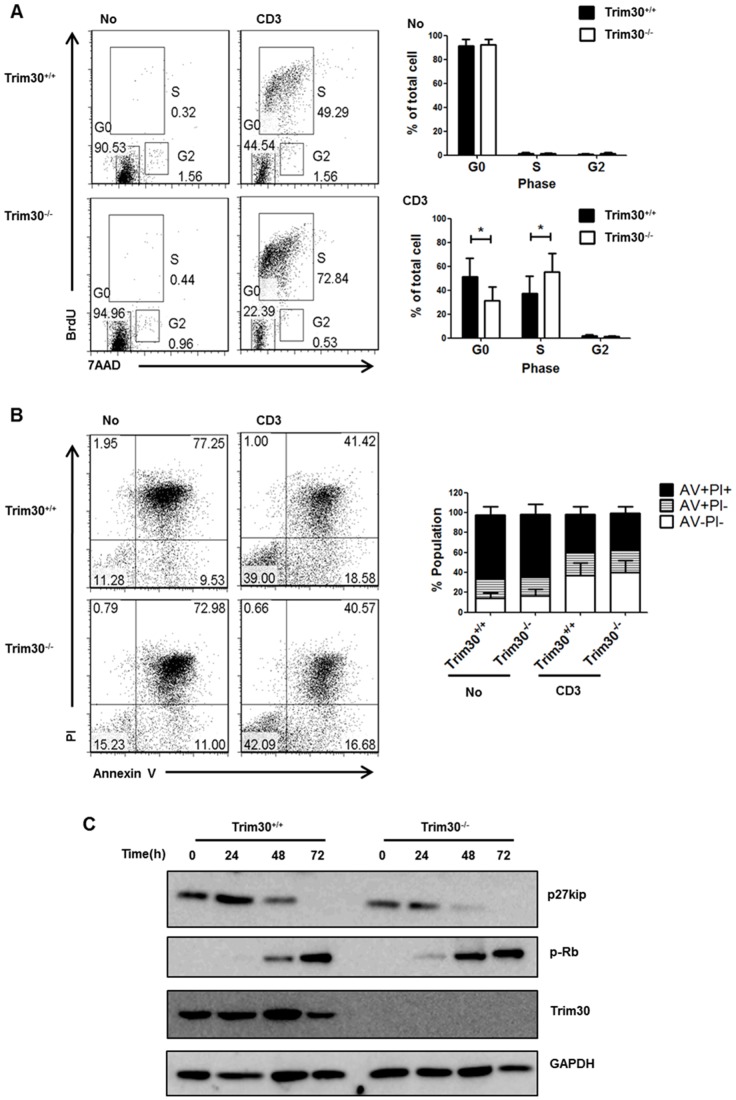
Modulation of the cell cycle in *Trim30* ^−/−^ CD4^+^ T cells. (**A**) The cell cycle profile of *Trim30* ^+/+^and *Trim30* ^−/−^ CD4^+^ T cells with (CD3) or without (No) anti-CD3 stimulation for 3 days. Each phase of the cell cycle was determined according to the levels of BrdU and 7AAD incorporated into the cells. The percentage of cells in each phase of the cell cycle is shown. The average of six independent experiments is shown error bars on the right. The *P*-values were calculated with a *t* –test. *, *P*<0.05. (**B**) Apoptotic ratio of *Trim30* ^+/+^ and *Trim30* ^−/−^ CD4^+^ T cells with (CD3) or without (No) anti-CD3 stimulation. The percentages of cells in early (annexin positive and PI negative) and late (double positive) apoptosis are given along with those of the viable cells (double negative). The average of five independent experiments is presented with error bars on the right. (**C**) Immunoblot analysis of cell cycle proteins in whole cell lysates of purified *Trim30* ^+/+^and *Trim30* ^−/−^ CD4^+^ T cells following stimulation with the anti-CD3 antibody (2 µg/ml) using the indicated antibodies for the indicated time period. Error bars in A and B denote s.d.

To assess the role of TRIM30 in CD4^+^ T cell proliferation, we analyzed the cell cycle progression of *Trim30* ^−/−^ CD4^+^ T cells. Upon CD3 stimulation for 72 h, both *Trim30* ^+/+^and *Trim30* ^−/−^ CD4^+^ T cells exhibited a sharp increase in BrdU and 7AAD incorporation, representing active DNA synthesis in S phase. However, more *Trim30* ^−/−^ (72.8%) than wild-type (49.3%) CD4^+^ T cells were found in S phase with a concomitant decrease in the corresponding cells in G0 phase (Figure 4A). To determine whether *Trim30* deletion has any effect on cell viability after TCR signaling, early and late apoptosis was analyzed by annexin V and PI staining (Figure 4B). CD3 stimulation sharply increased cell viability in both *Trim30* ^+/+^and *Trim30* ^−/−^ CD4^+^ T cells and resulted in no difference in the percentage of annexin V^+^PI^−^ cells (early apoptotic cells) or annexin V^+^PI^+^ cells (late apoptotic cells). Therefore, *Trim30* deficiency caused cell cycle hyper-progression into S phase but did not affect CD4^+^ T cell death.

We next investigated the expression of cell cycle regulators. Immunoblot analysis showed that in *Trim30* ^+/+^ CD4^+^ T cells, retinoblastoma (Rb) protein phosphorylation and p27kip expression were down- and up-regulated, respectively, 48 h after anti-CD3 stimulation (Figure 4C); however, *Trim30* ^−/−^ CD4^+^ T cells exhibited an earlier response in these expression patterns only 24 h after anti-CD3 stimulation. These results suggest that cell cycle progression in CD4^+^ T cells is indeed stimulated by the loss of TRIM30.

### Higher Proliferation of *Trim30* Knockout T cells in Rag1-deficient Mice

**Figure 5:**
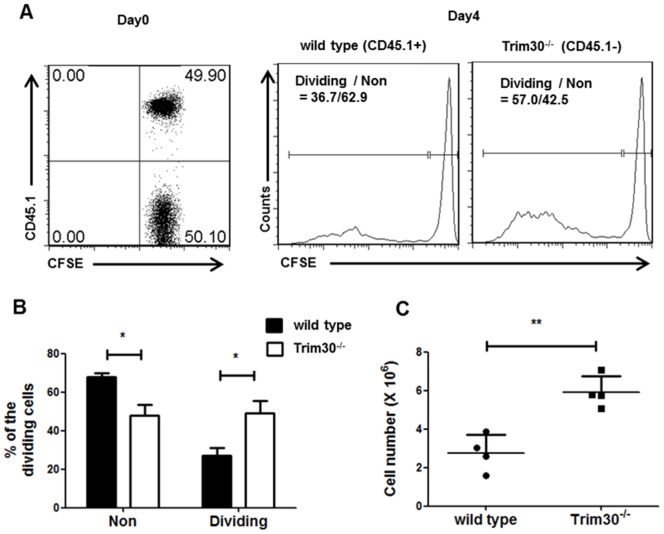
Trim30 is important for in^+^ T cell homeostasis. (**A**) CD4^+^ T cells isolated from CD45.2^+^ *Trim30* ^−/−^ and CD45.1^+^ wild-type congenic mice were labeled with CFSE and co-transferred to *Rag1* knockout mice. The dot-plot and histogram present the percentages of *Trim30* ^+/+^ (CD45.1 high) and *Trim30* ^−/−^ (CD45.1 low) donor CD4^+^ T cells on day 0 after adoptive transfer into the Rag1 knockout mice (left panel; day0). The wild-type (CD45.1^+^) and *Trim30* ^−/−^ (CD45.1^−^) CD4^+^ T cell distributions with the decreasing levels of CFSE intensities at day 4 after the adoptive transfer are shown on the right. The y axes in histogram plot represent the percentage of maximum counts. The percentages of proliferating cells are shown with those of the non-proliferating cells. (**B**) Average percentages of proliferating cells (Dividing) and non-dividing cells (Non) from three independent experiments are shown. (**C**) The numbers of adoptively transferred wild-type and *Trim30* ^−/−^ CD4^+^ T cells in recipient spleens after 4 days were measured from four individual mice. The *P*-value was calculated using a *t*–test. *, *P*<0.05; **, *P*<0.005. Error bars in B and C denote the s.d.

To confirm the physiological relevance of the enhanced proliferative phenotype of *Trim30* ^−/−^ T cells in vitro, we determined whether *Trim30* deletion influenced the homeostatic proliferation of T cells in lymphocyte-deficient mice. We purified CD4^+^ T cells from both *Trim30* ^−/−^ and wild-type mice, which expressed congenic markers CD45.2 and CD45.1, respectively, and labeled these cells with CFSE. In vitro-labeled wild type and *Trim30* ^−/−^ CD4^+^ T cells were mixed at a 1∶1 ratio and transferred to *Rag1* ^−/−^ recipient mice, which lack B and T cells (Figure 5A). The relative amounts of wild-type control and *Trim30* ^−/−^ splenic CD4^+^ T cells that were distinctly marked with the CD45.1^+^ and CD45.1^−^, respectively, were determined 4 days after the adoptive transfer. After selection of the adoptively transferred T cells using CD3 and CD4 markers, cell proliferation was analyzed by CFSE dilution (Fig. 5A). The competitive repopulation of adoptively transferred wild-type and *Trim30* ^−/−^ T cells revealed that the proliferating populations contained a significantly greater proportion of *Trim30* ^−/−^ T cells compared to *Trim30* ^+/+^ cells. Thus, twice the number of *Trim30* ^−/−^ T cells was detected in the recipient *Rag1* ^−/−^ mice after 4 days compared to wild-type T cells (Fig. 5B–C). These findings confirmed the negative function of TRIM30 in lymphopenia-induced T cell proliferation.

### Reduced Effector Function of *Trim30^−/−^* CD4^+^ T cells

**Figure 6:**
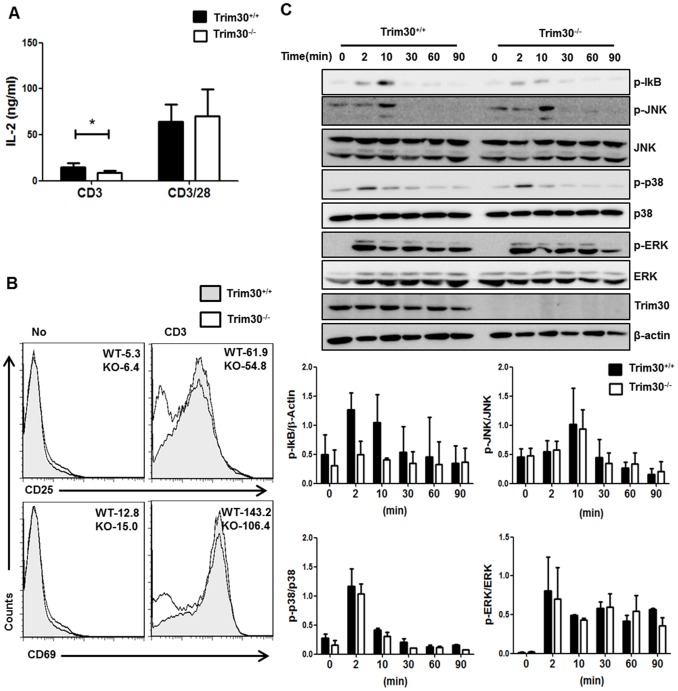
Effects of *Trim30* deficiency on classical TCR signaling. (**A**) Primary CD4^+^ T cells from *Trim30* ^+/+^and *Trim30* ^−/−^mice were stimulated with the indicated antibodies for 3 days, and then the supernatants were collected and assayed for IL-2 production by ELISA. Concentration of IL-2 is shown for three independent experiments. Error bars represent s.d. (**B**) The numbers of CD25− and CD69− expressing CD4^+^ T cells from *Trim30* ^+/+^ (gray area) and *Trim30* ^−/−^ (white area) mice 24 h after treatment with anti-CD3 (2 µg/ml) antibody are shown. Mean fluorescence intensity (MFI) values are given. (**C**) Purified CD4^+^ T cells from *Trim30* ^+/+^ and *Trim30* ^−/−^ mouse spleens were stimulated with anti-CD3 antibody (2 µg/ml) and anti-IgG (10 µg/ml) for the times indicated at the top. Lysates from CD4^+^ T cells were analyzed by immunoblotting using the indicated antibodies. The bar graph shows the averages of the band intensities. Data are representative results of at least three independent experiments. Error bars in A and C denote the s.d.

The above results indicate that TRIM30 raises the activation threshold required for T cell proliferation. Therefore, in the absence of *Trim30*, T cells began to proliferate in response to even weak activation signals from the TCR. Because TCR engagement promotes effector function as well as proliferation, we also analyzed the defects caused by *Trim30* deletion on T cell effector functions under the same conditions. Contrary to our expectations, *Trim30* ^−/−^ CD4^+^ T cells produced consistently lower levels of IL-2 compared to *Trim30* ^+/+^ CD4^+^ T cells upon stimulation with anti-CD3 antibody (Figure 6A). In addition, expression of CD25 and CD69, which mark early T cell activation, was also reduced in *Trim30* ^−/−^ CD4^+^ T cells compared to wild-type CD4^+^ T cells (Figure 6B); however, these defects were greatly diminished when cells were strongly stimulated with anti-CD3 and anti-CD28 agonists. Therefore, *Trim30* deletion enhanced T cell proliferation but resulted in a higher threshold for activation of effector function via TCR activation.

We then investigated the mechanisms underlying this enhanced CD4^+^ T cell proliferation and decreased effector function in *Trim30* ^−/−^ mice. We stimulated CD4^+^ T cells for 90 min with anti-CD3 antibodies and then examined cell lysates for phosphorylation of classical TCR signaling molecules. Activation of classical TCR signaling pathways, including the ERK, JNK, and p38 kinases, appeared similar in *Trim30* ^−/−^ and *Trim30* ^+/+^ CD4^+^ T cells, although phosphorylation of IκB was lower in *Trim30* ^−/−^ CD4^+^ T cells (Figure 6C). These observations suggest that TRIM30 is required for NF-κB activation following TCR activation with anti-CD3 agonist and that this effect occurs without enhancing proliferation during TCR activation in the absence of costimulatory signals.

## Discussion

TRIM30 is the mouse homolog of human TRIM5α protein, which exerts an anti-retroviral function in various cell types; however, to date, the role of TRIM30 in mice has been linked only with innate immune responses in macrophages and dendritic cells [15], [16], [20]. In this report, we established TRIM30 as a key regulator of TCR-induced signaling in CD4^+^ T cells. A lack of TRIM30 caused increased proliferation and cell cycle progression upon TCR cross-linking with anti-CD3 agonist. Furthermore, TRIM30 deficiency led to a high CD4/CD8 ratio in aged mice and increased expansion of adoptively transferred CD4^+^ T cells in lymphopenic hosts. Another interesting feature of *Trim30* ^−/−^ CD4^+^ T cells is the relatively lower effector function upon CD3 ligation, despite a highly proliferative phenotype. Thus, suppression of T cell proliferation concomitant with promotion of the effector function via TRIM30 may be required to prevent abnormal proliferation of T cells in the absence of optimal T cell activation signals, allowing adequate time for cells to properly differentiate into the correct T cell types before full activation.

*Trim30* ^−/−^ CD4+ T cells show the enhanced proliferation despite the reduced IL-2 production. We suggest that TRIM30 mediates T cell expansion through IL-2-independent proliferation. In vitro and in vivo studies present the evidence for IL-2-independent cell cycle regulatory mechanisms [21], [22]. These studies established that IL-2-independent mechanisms are involved in TCR/CD28 mediated cell cycle controlling [23], [24]. Reduced p27kip expression and enhanced phosphorylation of Rb in *Trim30* ^−/−^ CD4^+^ T cells suggest this possibility.

Recent studies showed that proliferation and effector function are regulated by different mechanisms [25], [26]. For example, the metabolic switch from oxidative phosphorylation to aerobic glycolysis is specifically required for effector function in T cells but not for proliferation [25]. On the other hand, a high multiplicity of TCR ITAM is required for T cell proliferation, whereas a low multiplicity of CD3 ITAM was sufficient to lead to cytokine secretion [26]. Therefore, activation of effector functions and proliferation upon TCR stimulation can be regulated independently.

Although both CD4^+^ and CD8^+^ T cells express TRIM30, our results show that the hyper-proliferation caused by *Trim30* deletion is only appear in CD4^+^ T cells, but not in CD8^+^ T cells. Because purified CD4^+^ T cells by using CD4 T Cell Isolation Kit contain Treg cells, the possibility of non-autonomous roles of Trim30 in Treg cells still exist. The percentage of Treg cells (CD25^+^Foxp3^+^) in splenic CD4^+^ T cells was 9 to 10%, but there was no difference in their abundance between Trim30^+/+^ and Trim30^−/−^ mouse (data not shown). It is necessary to follow-up studies addressing the roles of Trim30 in Treg and CD8^+^ T cells, albeit not to proliferation mechanism.

Notably, the effects of TRIM30 deficiency were apparent only when cells were stimulated with weak T cell activation signals. T cells should be resistant to activation following weak stimulatory signals in order to avoid hyperactivation of immune responses. We propose that TRIM30 regulates the threshold to effector function and proliferation in the presence of incomplete T cell stimulation; although, strong stimuli may override the regulatory activity of TRIM30 and induce TCR-mediated T cell activation.

In contrast to previous reports on *Trim30* knockdown or overexpression in monocytes [15], [16], we observed reduced NF-κB activation in *Trim30* ^−/−^ CD4^+^ T cells. This discrepancy may result from differences in the experimental conditions used in these studies (cell line-based *Trim30* knockdown vs. *Trim30* knockout mouse) or from difference in the tissue types (macrophage vs. T cells). Intriguingly, similar differences were also observed in the analysis of TAB2 and TAB3 mutant mice. TAB2 and TAB3, which are involved in NF-κB activation in monocytes (21), were reported as putative TRIM30 targets in knockdown experiment (13); however, TAB2 knockout mice have normal IL-1β production in response to IL-1R signaling [27]. A recent study of TAB2 and TAB3 knockout mice revealed that TAB2 and TAB3 are dispensable for activation of NF-κB and MAPKs in macrophages and MEFs [28]. Instead, TAB2 and TAB3 were shown to have critical role to control B cell activation by specifically regulating MAPKs [28]. Although the exact role of TRIM30 remains to be elucidated, our data showed that TRIM30 is required for optimal effector function of CD4^+^ T cells via NF-κB activation and concomitantly negatively regulates the proliferation of these T cells.

## Materials and Methods

### Ethics Statement

All animal experiments were performed in accordance with the Korean Food and Drug Administration (KFDA) guidelines. Protocols were reviewed and approved by the Institutional Animal Care and Use Committee (IACUC) of the Yonsei Laboratory Animal Research Center (YLARC) at Yonsei University (Permit Number: 2008-0012).

### Animal and Infection

All mice were maintained in the specific pathogen-free facility of the YLARC at Yonsei University following the IACUC guidelines. In experiments for survival study, male six- to ten-week-old littermate (22.9±1.6 g) *Trim30* ^+/+^ and *Trim30* ^−/−^ mice (n = 14∼18 per group) were used. Mice were challenged with LPS (20 mg/kg) or Listeria Monocytogenes (2×10^6^ CFU per mouse) by intraperitoneal (i.p) route. The experiment was repeated 3∼4 times, and data were pooled. The Kaplan-Meier method was used to plot survival curve. Survival and health status of infected mice were monitored daily. Mice reaching the humane endpoint (compound-related severe illness, as indicated by inability to eat and drink or a lack of response when gently prodded with a forceps) were euthanized by cervical dislocation under anesthesia with isoflurane. All mice were sacrificed by same method.

### Gene Targeting

*Trim30* knockout mice were generated using homologous recombination. A short (1.7 kb) and long (6.3 kb) arm were obtained from a C57BL/6J BAC clone (Children’s Hospital Oakland BACPAC Resources) using PCR. The following primers were used: short arm forward primer (5′-CCCATCGATAGACTTACACCAAGAT-3′), reverse primer (5′-GCGGAATTCTCAGCACTCACAGG-3′), long arm, forward primer (5′-CCCGAATTCACAGGGTACTTACC-3′), and reverse primer (5′- AAAGCGGCCGCCAGCATTTCTAA-3′). Products were ligated into the gene-targeting cassette vector pGK-TK-Neo and electroporated into 129/Sv E14 embryonic stem (ES) cells. Cells positive for homologous recombination were selected with G418 and ganciclovir and screened using Southern blotting. Positive ES cells were then injected into C57BL/6 blastocysts in the transgenic facility at the Ewha Laboratory Animal Genomic Center (Korea). Chimeras containing the target allele were backcrossed to C57BL/6 animals, and experiments were performed with mice that were backcrossed for more than 10 generations. For genotyping, genomic DNA was extracted from mouse tail biopsies, and PCR amplification was performed using the following primers: TRIM30 WT forward (5′-CAGGTGGAAAACCTCCACTTTTGC-3′), TRIM30 reverse (5′-CCCCATTGTTGAAAGTCACTAGATAGC-3′), and NEO KO forward (5′-GCCTTCTAGTTGCCAGCCATCTGTT-3′).

### Media, Reagents, and Antibodies

Lymphocytes were cultured in RPMI 1640 supplemented with 10% heat-inactivated FBS, 100 U/ml penicillin and streptomycin, 2 mM L-glutamine (Gibco), and 50 µM 2-mercaptoethanol (Sigmal-Aldrich, St. Louis, MO). The following reagents and antibodies were purchased from BD Pharmingen (San Jose, CA): 7AAD; annexin V; PI and anti-mouse CD4 (L3T4), CD8 (53-6.7), CD25 (7D4), CD69 (H1.2F3), CD44 (IM7), CD3ε (145-2C11) antibodies. In addition, the following reagents and antibodies were used: CFSE (Invitrogen, Carlsbad, CA), PMA (Sigma-Aldrich), and ionomycin (Sigma-Aldrich), as well as anti-TRIM30 (ab76953; Abcam, Cambridge, UK), anti-β-actin (4967; Cell Signaling, Danvers, MA), anti-GAPDH (sc25778; Santa Cruz, CA), and anti-IκB (sc371; Santa Cruz) antibodies. OVA peptide (323–339) was synthesized by AnyGen (Korea).

### Primary T Cell Culture, Proliferation Assay, and in vitro Differentiation

T cells were isolated from spleens using a CD4 T Cell Isolation Kit and a CD8 T Cell Isolation Kit (Miltenyi Biotech, Auburn, CA). T cells were labeled for 7 min with 1.25 µM CFSE, followed by termination with 1/10 volume of FBS. Cells were washed 3 times. Labeled cells were cultured in pre-coated plates with anti-CD3 (145-2C11) and soluble anti-CD28 antibodies (37.51) (BD). The BrdU Flow kit (BD) was used to identify cell cycle populations in vitro. BrdU was added to cell cultures with anti-CD3 and soluble anti-CD28 antibodies during the final 7 h of culture. For ova peptide stimulation, splenocytes from OT-II mice were cultured with OVA peptide (323–339).

### Flow Cytometry Analysis

We isolated mononuclear cells from thymus, spleen, and lymph nodes of *Trim30* ^+/+^ and *Trim30* ^−/−^ mice. These cells were stained with antibodies to surface antigens for 30 min at 4°C. After several washes, cytofix/cytoperm was added and incubated for 30 min at 4°C. CD4^+^ T cell apoptosis was assessed using annexin V-FITC and propidium iodide (PI) staining. Samples were loaded on a FACS Calibur, and data analyses were performed with Flow Jo software.

### Quantitative Real-time PCR

Total RNA was isolated using the Trizol reagent (Invitrogen) according to the manufacturer’s instructions. Then, cDNA was synthesized from 2 µg of the isolated RNA using SuperScript II Reverse Transcriptase (Invitrogen) with random hexamers or oligo-dT as the primers. After synthesis, cDNA was diluted 1∶10 in triple distilled water (TDW), and 3 µl was used for real-time PCR using a Bio-Rad CFX. Gene expression levels were normalized to *GAPDH* RNA levels. The following primers were used: *Trim30* forward primer (5′-TTCCAGAGGAGGAGCAGAAGGTG-3′), *Trim30* reverse primer (5′-TCACAAAGCCAGCAGATGACCATC-3), *GAPDH* forward primer (5′- GGCAAATTCAACGGCACAGTCAAG-3′), *GAPDH* reverse primer (5′- TCGCTCCTGGAAGATGGTGATGG-3′), *IL-6* forward primer (5′- TAAGCATATCAGTTTGTGGAC-3′), *IL-6* reverse primer (5′- TCATATTGTCAGTTCTTCGTAG-3′), *IL-12p40* forward primer (5′- GAAGTGATATTGATAAGAAACCAG-3′), *IL-12p40* reverse primer (5′- AAGCATATACCACCGATACC-3′), *TNFα* forward primer (5′- ATGTCCATTCCTGAGTTCTG-3′), *TNFα* reverse primer (5′- AATCTGGAAAGGTCTGAAGG-3′), *IFNα* forward primer (5′- AGGACTCATCTGCTGCATGGAATG-3′), *IFNα* reverse primer (5′- CACACAGGCTTTGAGGTCATTGAG-3′), *IFNβ* forward primer (5′- CCACTTGAAGAGCTATTACTG-3′), *IFNβ* reverse primer (5′- AATGATGAGAAAGTTCCTGAAG-3′).

### Adoptive Transfer of T cells

CD4^+^ T cells from spleens of 6- to 8-week-old CD45.1^+^ congenic C57BL/6 mice (wild-type) or CD45.2^+^ *Trim30* ^−/−^ mice were purified using CD4 (L3T4) MicroBeads (Miltenyi Biotech) and labeled with 5 µM CFSE. Then, 5×10^6^ wild-type and *Trim30* ^−/−^ CD4^+^ T cells (mixed at a ratio of 1∶1) were injected intravenously into sex- and age-matched Rag1^−/−^ mice. Mice were sacrificed 4 days after the transfer. CFSE dilutions patterns of cells from wild-type and *Trim30* ^−/−^ mouse spleens were measured by FACS.

### Statistical Analysis

GraphPad Prism 5.0 software was used for statistical analysis. An unpaired two-tailed t-test was used to compare experimental groups. Data shown in the figures represents the mean ± SD. Differences were considered statistically significant when the *P*-values were less than 0.05.

### Bone Marrow-derived Macrophage (BMDM) Isolation and Culture

For generating BMDMs, bone marrow cells were collected from femurs and tibias of *Trim30^+/+^ and Trim30^−/−^* mice by flushing with Dulbecco’s phosphate buffered saline (DPBS). BMDMs were differentiated for 6–7 days in DMEM (Invitrogen) supplemented with 20% FBS (Gibco), 50 U/ml penicillin, 50 µg/ml streptomycin, and 20% cultured supernatant from L929 cells.
