# Correction: Tripartite Motif-Containing Protein 30 Modulates TCR-Activated Proliferation and Effector Functions in CD4^+^ T Cells

**DOI:** 10.1371/journal.pone.0099267

**Published:** 2014-05-27

**Authors:** The PLOS ONE Staff

The image for Figure 1 was inadvertently published as a duplicate of Figure 2. Please view the correct image for Figure 1 here.

**Figure 1:**
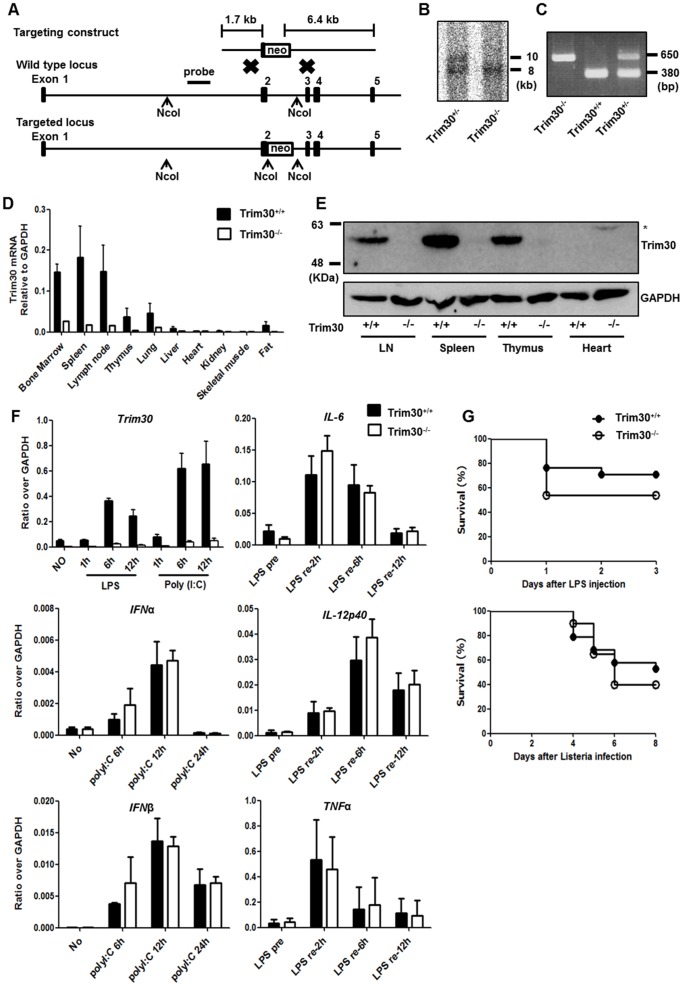
Generation of *Trim30* knockout mice. (**A**) A diagram representing the targeting construct, the *Trim30* gene locus (Wild-type locus), and the locus after targeting (Targeted locus). The targeting construct contains a stop codon and a neomycin selectable marker in exon 2 of *Trim30*. (**B**) Genomic DNA fragments from *Trim30* ^+/−^ and *Trim30* ^−/−^ progeny after Southern blotting with NcoI digestion. Wild-type alleles (10 kb) and the targeted alleles (8 kb) are indicated. (**C**) Genomic DNA isolated from *Trim30* ^+/+^, *Trim30* ^−/−^, and *Trim30* ^+/−^ was subjected to PCR. (**D**) Tissue *Trim30* mRNA expression from *Trim30* ^+/+^ and *Trim30* ^−/−^ mice. RT-PCR analysis revealed high *Trim30* transcript levels in lymphoid organs (spleen, thymus, and lymph node) and bone marrow in contrast to the low levels of *Trim30* transcripts in non-hematopoietic tissues (**E**) TRIM30 protein expression level in tissues from *Trim30* ^+/+^ and *Trim30* ^−/−^ mice as determined with immunoblotting using anti-Trim30 antibody. *, non-specific signal. (**F**) BMDMs were stimulated with LPS (200 ng/ml) or poly(I:C) (5 µg/ml), and *Trim30* transcripts were quantified by quantitative RT-PCR. For detection of cytokine expression, *Trim30* ^+/+^ and *Trim30* ^−/−^ BMDMs were pretreated for 18 hr with LPS (LSP pre) and then restimulated with LPS (LPS re) indicated time or stimulated with poly(I:C) and transcripts for indicated cytokines were quantified by quantitative RT-PCR. Expression was normalized to GAPDH. (**G**) Survival of mice (n  =  14 per group) given i.p injection of LPS (20 mg/kg) (upper panel). Survival of mice (n  =  18 per group) given i.p infection of Listeria monocytogenes (2×10^6^ CFU per mouse) (lower panel). Data are representative results from three independent experiments. Error bars in D, E, F indicate s.d.
